# Avoiding Diagnostic Pitfalls in Facial Pain: A Case Report of Paroxysmal Hemicrania Initially Treated as Trigeminal Neuralgia

**DOI:** 10.7759/cureus.117284

**Published:** 2026-10-01

**Authors:** Abdul Amir Chehimi, Marwan D Jaber, Ali Ba Nafea, Teimuraz Kakubava

**Affiliations:** 1 Neurology Department, Consilium Medulla Multiprofile Clinic, Tbilisi, GEO; 2 School of Health Sciences, University of Georgia, Tbilisi, GEO

**Keywords:** case report, diagnostic error, facial pain, headache diary, ichd-3, indomethacin, paroxysmal hemicrania, trigeminal autonomic cephalalgia, trigeminal neuralgia, verapamil

## Abstract

A 21-year-old man presented with a two-year history of recurrent, strictly unilateral left-sided pain in the upper maxillary and orbital regions, radiating to the temporal and occipital areas. Individual attacks lasted 20 to 30 minutes and occurred 10 to 20 times daily, on most days throughout the two-year period. He had been diagnosed elsewhere with trigeminal neuralgia and treated with carbamazepine 200 mg twice daily without benefit. He denied photophobia, phonophobia, and ipsilateral cranial autonomic features, but described prominent restlessness during attacks. No cutaneous or mucosal trigger could be identified, and neurological examination was normal. The attack duration, the reported high attack frequency, the absence of tactile triggers, and the restlessness were inconsistent with classical trigeminal neuralgia and raised suspicion of a trigeminal autonomic cephalalgia, including probable paroxysmal hemicrania. Carbamazepine was withdrawn, and an escalating oral indomethacin trial was initiated, reaching 225 mg daily and maintained at that dose for 14 consecutive days. A headache diary begun on the first day of the trial recorded headache on all 20 days, and the patient reported no improvement at any point. Because the phenotype fulfilled all but the indomethacin-response criterion, the diagnosis was recorded as probable paroxysmal hemicrania, while cluster headache and other trigeminal autonomic cephalalgias remained in the differential diagnosis. Verapamil 240 mg daily was substituted on day 21. Headache days fell to 23 of 31 in the first month and 13 of 31 in the second, with the last 18 days of the second month free of headache. At telephone follow-up, the patient reported sustained resolution, with only mild headaches related to sleep deprivation that he considered distinct from his previous syndrome. He remains on verapamil. Whether verapamil produced the improvement cannot be established from a single uncontrolled case, as the drug was never withdrawn, and spontaneous remission remains possible. This case illustrates the clinical overlap between trigeminal neuralgia and the trigeminal autonomic cephalalgias and highlights the value of structured longitudinal symptom recording, ideally including individual attack count, duration, autonomic features, and triggers.

## Introduction

Facial pain is a frequent presenting complaint in the outpatient setting and carries a wide differential diagnosis that includes dental, musculoskeletal, neuropathic, vascular, and primary headache conditions. Trigeminal neuralgia is among the most frequently considered causes of paroxysmal facial pain and arises from abnormal irritation of the trigeminal nerve. It is defined by short-lasting, unilateral, stabbing, or electric shock-like pain along the affected trigeminal divisions, typically provoked by innocuous stimuli such as talking, swallowing, touching the face, or brushing the teeth. Other short-lasting headache disorders, including the trigeminal autonomic cephalalgias (TACs) such as paroxysmal hemicrania and short-lasting unilateral neuralgiform headache attacks with conjunctival injection and tearing (SUNCT), can be mistaken for trigeminal neuralgia when the pain falls within the trigeminal distributions [1,2].

Paroxysmal hemicrania is an uncommon primary headache disorder within the spectrum of TACs. It manifests as repeated attacks of severe, strictly unilateral pain over the orbital, supraorbital, temporal, or facial regions, usually accompanied by ipsilateral cranial autonomic features such as lacrimation, conjunctival injection, nasal congestion or rhinorrhea, eyelid edema, and ptosis. Under the International Classification of Headache Disorders Third Revision (ICHD-3) classification, attacks last 2 to 30 minutes and recur many times per day, and a definite diagnosis requires that they be absolutely prevented by therapeutic doses of indomethacin [1,3].

Despite these features, paroxysmal hemicrania is often missed in practice because of its rarity and its phenotypic overlap with other unilateral facial and head pain syndromes. A systematic review and meta-analysis estimated its relative frequency at approximately 0.3% among adults evaluated for headache in tertiary care [4]. Paroxysmal hemicrania also does not always present with a textbook history. Cranial autonomic features are characteristic but are not strictly required, as ICHD-3 permits a sense of restlessness or agitation to satisfy the same criterion, and their absence or equivocal presentation can obscure the diagnosis. Descriptions of the pain as stabbing, shooting, burning, or electric may further lead a clinician toward trigeminal neuralgia without full consideration of a trigeminal autonomic headache [5,6].

The distinction carries direct therapeutic consequences. Carbamazepine and oxcarbazepine are the established first-line treatments in classical trigeminal neuralgia, whereas indomethacin is the first-line agent in paroxysmal hemicrania [2,5]. In the present case, the distinction had direct consequences, as the patient continued an ineffective drug for a month before the phenotype was reassessed. The temporal profile of attacks, their frequency and distribution, the presence or absence of cranial autonomic features, and the response to an adequate indomethacin trial should therefore be elicited in any patient presenting with recurrent unilateral facial pain, as indomethacin responsiveness is characteristic enough to form part of the diagnostic criteria for paroxysmal hemicrania [1,3,5].

These considerations apply with particular force to a patient already labelled as having trigeminal neuralgia who has an atypical attack profile, an unexpected response to treatment, or associated features that do not fit. Reassessment of the phenotype may reveal a different headache disorder, which is one reason to keep a broad differential in refractory or atypical facial pain. MRI of the brain is commonly obtained in newly diagnosed paroxysmal hemicrania to exclude structural or secondary causes that can mimic the syndrome, although the extent of investigation depends on the clinical context [3,5].

This report describes a patient whose initial diagnosis of trigeminal neuralgia did not fit the reported phenotype on re-evaluation and whose presentation was more suggestive of paroxysmal hemicrania. The case illustrates how closely these conditions can resemble one another, and how attack duration, attack frequency, cranial autonomic features, and the response to indomethacin bear on the distinction.

## Case presentation

Patient information

A 21-year-old man with no known chronic medical conditions or drug allergies presented to the neurology service at Consilium Medulla Multiprofile Clinic in March 2026 for evaluation of recurrent facial and orbital pain.

Clinical findings

The patient reported an approximately two-year history of recurrent, strictly unilateral left-sided pain centered on the upper maxillary and orbital regions and radiating to the temporal and occipital areas. Individual attacks lasted 20 to 30 minutes and occurred 10 to 20 times daily. Attacks had been present on most days throughout the two-year period, with no remission lasting three months or longer.

Approximately one month before presentation, he had been evaluated by a neurologist, diagnosed clinically with trigeminal neuralgia, and started on carbamazepine 200 mg twice daily. Because his symptoms persisted, brain MRI was obtained, and he was referred for further neurological evaluation.

He reported taking it without dose change from the time it was prescribed until his presentation to us, and reported no adverse effects. Whether the dose had been titrated or adherence formally assessed by the referring clinician could not be established, and the dose reached was below the range often required in trigeminal neuralgia, so the absence of benefit should be interpreted with that in mind.

At presentation, vital signs were normal: blood pressure of 100/70 mmHg, heart rate of 73 beats/minute, respiratory rate of 15 breaths/minute, and temperature of 36.5 °C. Neurological examination was normal. He was alert and fully oriented, with normal speech and cognition. Cranial nerves II to XII were intact, pupils were equal and reactive to light, extraocular motility was normal, facial movements were symmetric, and facial sensation across the ophthalmic, maxillary, and mandibular divisions of the trigeminal nerve was preserved bilaterally, with intact corneal reflexes. Motor examination showed normal bulk and tone with 5/5 strength throughout, symmetric deep tendon reflexes, and bilateral flexor plantar responses. Sensory examination and coordination were normal, without dysmetria. Gait and tandem gait were normal, and Romberg testing was negative. No focal neurological deficit was identified.

On systematic headache assessment, he denied photophobia and phonophobia and reported no ipsilateral cranial autonomic manifestations during attacks, including conjunctival injection, lacrimation, nasal congestion, rhinorrhea, eyelid edema, forehead or facial sweating, miosis, and ptosis. He did describe prominent restlessness and agitation during attacks. He identified no cutaneous or mucosal trigger, and the attacks were not provoked by touching the face, chewing, or speaking.

Diagnostic assessment

The patient’s symptoms were evaluated against the ICHD-3 criteria, with migraine, tension-type headache, cluster headache, trigeminal neuralgia, SUNCT, short-lasting unilateral neuralgiform headache attacks with cranial autonomic symptoms (SUNA), hemicrania continua, and medication-overuse headache considered as differential diagnoses. Symptomatic analgesic use was intermittent and did not reach the frequency or regularity required for a diagnosis of medication-overuse headache.

The brain MRI had been performed before referral, and the images were not available to us. The radiology report was reviewed and described no acute focal intracranial lesion. This does not amount to a normal study or to exclusion of all secondary causes of headache, and the images were not available for independent review. Bilateral type II anterior inferior cerebellar artery loops were reported. Based on the report alone, and given their bilateral distribution in a patient with strictly unilateral pain and no audiovestibular symptoms, they were considered unlikely to be related to the headache syndrome.

The combination of recurrent, severe, short-lasting orbital and maxillary pain, high attack frequency, and prominent restlessness, despite the absence of cranial autonomic features, was more suggestive of a TAC, and of paroxysmal hemicrania in particular, than of trigeminal neuralgia. The absence of autonomic signs did not exclude the diagnosis, because ICHD-3 criterion C is satisfied by either at least one ipsilateral cranial autonomic feature or a sense of restlessness or agitation. A working diagnosis of paroxysmal hemicrania was therefore made, to be confirmed or refuted by a therapeutic trial of indomethacin.

Intervention

Carbamazepine was tapered rather than stopped abruptly, concurrently with the first part of the indomethacin trial. The exact taper schedule was not documented. The patient was given a headache diary in which to record the details of his symptoms. An escalating oral indomethacin trial was then initiated (Table 1).

**Table 1:** Escalating oral indomethacin trial.

Treatment period	Dose	Total daily dose
Days 1–3	25 mg three times daily	75 mg/day
Days 4–6	50 mg three times daily	150 mg/day
Days 7–20	75 mg three times daily	225 mg/day

The 225 mg/day dose reached the maximum specified by ICHD-3 for a therapeutic indomethacin challenge and was maintained for 14 consecutive days. The headache diary was started on the first day of the trial. The patient recorded headache on all 20 days of the trial and reported no improvement at any point. Verapamil 240 mg/day was substituted on day 21.

Follow-up and outcomes

During the first month of verapamil therapy, headache occurred on 23 of 31 days, leaving eight headache-free days, with mild-to-moderate pain intensity. During the second month, headache occurred on 13 of 31 days, all within the first half of the month, giving 18 consecutive headache-free days at the end of the recording period, with intensity again mild to moderate.

The patient stopped recording the diary after approximately two months of verapamil therapy, as attacks had become infrequent and were no longer troubling him. Diary completion was checked at each telephone contact, when the recorded entries were reviewed with him, but the diary captured headache days only and did not record individual attack counts or attack durations. He was contacted by telephone on three occasions after the diary ended, in June, July, and August 2026. On each occasion, he reported that the short, severe, high-frequency attacks that had characterized his original syndrome no longer occurred. He continued to describe occasional mild, nonspecific headaches associated with sleep deprivation, which differed from his previous attacks in character, severity, and frequency, and which he did not regard as part of the same condition. Adherence and tolerability were assessed by structured telephone interviews throughout, and he reported full adherence to both drug trials and no adverse effects. He remains on verapamil 240 mg/day. At the most recent contact, approximately three months after the last attack recorded in the diary, he remained free of the original attacks.

The final diagnosis remained probable paroxysmal hemicrania, as an absolute response to indomethacin is required for a definite diagnosis.

## Discussion

This patient was treated for trigeminal neuralgia for a month. Based on the history obtained at our assessment, the reported attack duration, frequency, and lack of tactile triggers were atypical for classical trigeminal neuralgia. When he reached us, his attacks fulfilled all but the indomethacin response criterion for paroxysmal hemicrania. He improved after verapamil was substituted and has remained well. The first part of that sequence is a problem of clinical reasoning. The second is a problem for the classification.

A phenotype that fits trigeminal neuralgia poorly

The site of the pain was compatible with trigeminal neuralgia, but little else in the history we obtained was. ICHD-3 requires paroxysms lasting from a fraction of a second to two minutes; pain that is electric shock-like, stabbing, or sharp; and attacks provoked by innocuous stimuli within the affected trigeminal distribution [1]. Our patient’s attacks lasted 20 to 30 minutes. He reported 10 to 20 of them on a typical day, and nothing he touched or did brought them on. Duration alone separates the two conditions, seconds to two minutes in trigeminal neuralgia against 2 to 30 minutes in paroxysmal hemicrania, and it remains one of the most reliable discriminators at the bedside [1,6].

Treatment gave a second signal, though a weaker one. Carbamazepine is the standard first-line treatment for trigeminal neuralgia, and one of the characteristic features of the disorder is that the pain responds to it, at least in its early stages [1]. He took carbamazepine 200 mg twice daily for about a month without benefit. Response to treatment is not a diagnostic criterion, however, and non-response cannot exclude trigeminal neuralgia when the dose reached was modest and adherence was not formally assessed. It prompted reassessment rather than settling anything.

How the initial diagnosis may have been reached

We never saw the first clinician’s reasoning, so what follows is a hypothesis. It is at least a familiar one. The loudest feature of this presentation is facial pain in the maxillary and orbital regions, and trigeminal neuralgia is the label that attaches most easily to recurrent unilateral facial pain. Viana and colleagues, reviewing diagnostic and therapeutic errors across the TACs, found that trigeminal neuralgia is the first condition many non-headache specialists consider when faced with a recurrent facial pain syndrome, for reasons that are not clear; they cite a series in which 16% of cluster headache patients had carried that label first [6]. Paroxysmal hemicrania is missed in much the same way. It is usually mistaken for dental disease or temporomandibular disorder, sometimes for trigeminal neuralgia, and the risk is highest in the roughly 10% of patients whose attacks can be set off by neck flexion or pressure over the C2 root [6].

Put in general terms, the pattern of reaching for the diagnosis that matches the site of pain rather than its temporal profile is one that the literature on diagnostic reasoning describes as anchoring, in which an early salient feature is fixed upon at the expense of information arriving later, followed by premature closure, in which a working diagnosis is accepted before it has been checked [7]. A month of continued treatment without reassessment would be described as diagnostic momentum. We cannot say that this is what occurred here, as we never saw the original assessment or the reasoning behind it. We raise it because the pattern is a recognized one in facial pain, and because what separates these conditions is how long the attacks last and how often they come, not where in the face they are felt.

The imaging finding

MRI reported bilateral type II anterior inferior cerebellar artery loops. Under the Chavda classification, a type II loop enters the internal auditory canal but does not extend beyond half its length [8]. Loops like this are common in people with no symptoms. In a consecutive series of adults imaged with three-dimensional constructive interference in steady-state sequences and screened to exclude audiovestibular symptoms, anterior inferior cerebellar artery loops were found on about 47% of sides, type II being the second most frequent after type I [8]. The anatomy also argues against a causal role. It runs with the seventh and eighth cranial nerves at the cerebellopontine angle, not with the trigeminal nerve. Here it was bilateral, in a man with strictly unilateral pain, normal facial movement, and no hearing or balance complaint. It therefore provides no support for a diagnosis of trigeminal neuralgia. We recorded it as an incidental variant, and it played no part in our reasoning. Neurovascular contact is a frequent MRI finding in people without symptoms; it means something only when the clinical picture fits it.

Fitting the phenotype to paroxysmal hemicrania

Against ICHD-3, the reported phenotype met criteria A to D for paroxysmal hemicrania. The pain was severe and strictly unilateral, centered on the orbital and maxillary regions and radiating to the temporal and occipital areas. Attacks lasted 20 to 30 minutes, and he reported 10 to 20 of them daily, which, if accurate, is well beyond the requirement of more than five per day for over half of the active period [1]. This figure was obtained by interview and was not counted prospectively. Frequencies of this order are ordinary in this disorder rather than exceptional: mean attack frequency in the largest prospective series was 11 per day [9], and counts as high as 40 daily have been reported. The reported course was unremitting. He had attacks on most days for two years, with no remission approaching the three months that would define an episodic pattern, so the reported pattern corresponds to the chronic rather than the episodic form [1]. The maxillary and occipital involvement deserves comment, because the site specification in the criteria is narrower than what is seen in practice. In the largest prospective series, pain was orbital and temporal in 77% of patients but was also reported in the second and third trigeminal divisions, the occiput, the vertex, the jaw, the ear and the neck; the authors proposed removing the specification of the attack site from the criteria altogether [9].

The absence of cranial autonomic features is the part that complicates classification, though it does not exclude the diagnosis. Criterion C is met by either one ipsilateral cranial autonomic feature or a sense of restlessness or agitation [1]. Our patient described marked restlessness at every consultation. Agitation is not a fringe feature here: 80% of patients in the Cittadini et al. series were restless, agitated, or both during attacks [9]. Some earlier accounts describe patients with paroxysmal hemicrania as comparatively quiet, in contrast to cluster headache [6]. The two accounts disagree, which is reason enough not to lean on behavior during attacks when separating these conditions. Cases without autonomic features have been described directly. Prakash and colleagues, reviewing 22 consecutive patients, found individuals who met every criterion but one, two of them with no autonomic feature at all, and argued that the criteria need revision [10].

Differential diagnosis within the trigeminal autonomic cephalalgias

Cluster headache was the principal alternative, and the later response to verapamil kept it under consideration. Two features that might appear to separate the two conditions do not. The absence of cranial autonomic manifestations does not, because ICHD-3 allows either an ipsilateral autonomic feature or restlessness for cluster headache exactly as it does for paroxysmal hemicrania, so our patient’s restlessness satisfies criterion C for both [1]; in a series comparing 54 patients with cluster headache and eight with paroxysmal hemicrania, restlessness was recorded in 57% and 63%, respectively, with no significant difference, although the paroxysmal hemicrania group was small [11]. Attack duration does not separate them either. Twenty to 30 minutes falls at the short end of the 15 to 180 minutes described for cluster headache while sitting comfortably within the two to 30 minutes of paroxysmal hemicrania [1], and the two disorders overlap considerably in their clinical characteristics [11].

Attack frequency is the feature that most nearly separates them, though not decisively. ICHD-3 sets the range for cluster headache at one attack every other day to eight per day [1]. The reported 10 to 20 attacks daily would exceed that ceiling and fall within the range expected in paroxysmal hemicrania, where mean attack frequency is higher and mean attack duration shorter than in cluster headache [9,11]. As that figure rests on recall rather than a prospective count, it cannot carry the distinction on its own. The temporal pattern points the same way. His reported course was unremitting over two years, and the chronic unremitting form predominates in paroxysmal hemicrania whereas cluster headache is episodic in the great majority of patients; in the comparative series above, the episodic to chronic ratio among cluster headache patients was 4.4 to 1 [11]. We should nevertheless be explicit that three features of this case run the other way: his sex, as cluster headache is male-predominant while paroxysmal hemicrania is not; the failure of indomethacin; and the improvement on a drug that is first-line for cluster headache. Attack frequency was also taken from clinical interview rather than counted prospectively, so the figure that decides the question is the one we were least able to verify.

The remaining differentials were considered less likely, though none can be excluded with certainty on history alone. Short-lasting unilateral neuralgiform headache attacks with conjunctival injection and tearing, and the SUNA variant, were thought unlikely based on attack duration, measured in seconds to a few minutes, and by the absence of the conjunctival and lacrimal features that define the former [1]. Hemicrania continua was thought unlikely because he reported no continuous background headache between attacks, though patients do tend to report only their worst pain and to leave out a persistent low-grade component unless asked directly [6]. Migraine was not supported: no aura, no consistent nausea or vomiting, no photophobia or phonophobia, and no reliable worsening with routine activity. Medication-overuse headache was considered, as he had headache on most days over a long period, but his use of symptomatic medication was intermittent and did not reach the frequency or regularity that ICHD-3 requires [1].

The indomethacin problem

Criterion E for definite paroxysmal hemicrania requires that attacks be prevented absolutely by therapeutic doses of indomethacin [1]. Our patient completed an escalating oral trial to 225 mg daily and stayed at that dose for 14 days. This reaches the maximum dose specified in the classification: an initial dose of at least 150 mg daily orally, increased to 225 mg if required [1]. The headache diary was begun on the first day of the trial, and he recorded headache on all 20 of those days. He reported no improvement at any point. The diagnosis could therefore go no further than probable paroxysmal hemicrania, coded under probable TAC, which applies when all but one of criteria A to E are met, and no other ICHD-3 diagnosis fits better [1].

An adequate negative indomethacin test requires that a therapeutic dose be reached, sustained long enough for a response to declare itself, and actually absorbed and taken. The first two conditions were met, as response in paroxysmal hemicrania typically occurs within days of reaching an effective dose and he remained at 225 mg daily for 14 days. The third could not be verified.

Adherence, tolerability, and the absence of side effects were established only by structured telephone interview. There were no pill counts, no serum concentrations, no supervised dosing, and no parenteral challenge. He said he took the drug as prescribed and had no adverse effects, which is consistent with an adequate trial but does not demonstrate one. Apparent unresponsiveness that turns out to be intolerance is a recognized trap and can throw unwarranted doubt on a sound diagnosis [12]. A supervised parenteral indomethacin challenge, given intramuscularly under placebo control, was developed for exactly this ambiguity in paroxysmal hemicrania and hemicrania continua and could have been considered to clarify treatment responsiveness [13]. It was not available in this setting. We therefore cannot exclude definite paroxysmal hemicrania. We call the diagnosis probable because of what we could not verify, not because we showed that he failed to respond to indomethacin. The wider literature offers little help: there is no guidance for patients who cannot take indomethacin or do not respond to it, and the evidence for every alternative rests on small series and individual reports [14].

The response to verapamil, and its alternative explanations

Verapamil 240 mg daily replaced indomethacin on day 21. Blood pressure and pulse recorded at the initial consultation were 100/70 mmHg and 73 beats/minute. No electrocardiogram was obtained before or during verapamil therapy, and because all subsequent contact was by telephone, no follow-up blood pressure, pulse, or PR interval could be assessed. This represents a monitoring limitation, as verapamil can cause bradycardia and atrioventricular conduction delay, and cardiovascular and electrocardiographic monitoring is recommended before initiation and with dose escalation. The dose used here remained at the lower end of the range and was not escalated, and no cardiac symptoms were reported at any point. Headache days fell from 20 of 20 during the indomethacin trial to 23 of 31 in the first month of verapamil and 13 of 31 in the second. The last 18 days of the second month were free of headache, and the remission has held. Verapamil has been reported as a second-line option here, on a limited evidence base. Evers and Husstedt, in a prospective open trial in 10 patients with chronic paroxysmal hemicrania, concluded that acetylsalicylic acid and verapamil were the most effective second-choice drugs [15]. Individual reports also describe benefit from calcium channel blockers in episodic paroxysmal hemicrania [16]. All of the evidence for alternatives to indomethacin rests on small open series and single cases [14].

There are other explanations for what happened, and we cannot rule them out. The pattern was a step down in the first month after the switch and a further fall in the second, with remission at about 10 weeks. The timing is compatible with a treatment effect, but it is equally compatible with coincidence, and the interval to full remission is longer than verapamil usually takes to work. His reported course had been unremitting rather than episodic, which would make a coincidental remission less likely, but the pattern rests on history rather than prospective observation. Chronic paroxysmal hemicrania can still remit spontaneously, and a slow decline ending in freedom from attacks is what natural fluctuation would look like. He remains on verapamil, so there has been no dechallenge or rechallenge, and the timing cannot be separated from coincidence. Response to treatment is a weak diagnostic instrument in any case, and we have not used the verapamil response to argue for or against any classification. What we can report is the sequence. The attacks had run for two years and had not responded to two previous drugs. They stopped after verapamil was started and have not come back. We cannot show that the verapamil is what stopped them.

Delay, access, and the conditions of follow-up

He waited about two years from onset before seeking specialist assessment. Long delays are the rule in this group of disorders rather than the exception. In cluster headache, the best studied of the TACs, a systematic review and meta-analysis covering 8,654 patients put the pooled diagnostic delay at 10.4 years [17]. The rarer indomethacin-responsive syndromes do no better [6].

This patient’s course differed in one further respect. He never came back to the clinic after the first consultation, which was provided without charge, and every later assessment was made by telephone. Cost was part of the reason. We still completed ICHD-3 phenotyping, an escalating drug trial, a prospective headache diary, and longitudinal follow-up. We include these details because case reports rarely say how the follow-up was actually carried out. Telephone follow-up allowed longitudinal symptom reporting in this case, but it could not replace in-person reassessment, objective safety monitoring, or prospective attack-level documentation, and it should not be taken as a substitute for them where in-person review is possible.

Strengths and limitations

The strengths are the diary kept prospectively across the indomethacin trial and two months of verapamil therapy rather than a retrospective reconstruction, phenotyping against explicit ICHD-3 criteria with the main differential diagnoses considered and excluded, an indomethacin trial taken to an adequate dose and duration rather than abandoned early, and follow-up long enough to show that the change held.

The limitations are substantial. This is one patient. Adherence to indomethacin, tolerability, and the absence of adverse effects rest entirely on self-report, and no parenteral indomethacin test was performed, so definite paroxysmal hemicrania can be neither established nor excluded. Carbamazepine was tapered concurrently with the early part of the indomethacin trial and the exact schedule was not documented, although the patient had already taken carbamazepine for a month without benefit. Attack frequency, the feature that most clearly separates paroxysmal hemicrania from cluster headache in this case, was obtained by clinical interview and never counted prospectively; the diary recorded headache days rather than individual attacks, so although it documented headache on every day of the indomethacin trial, it could neither confirm nor refute the 10 to 20 daily attacks he described. The diary also recorded duration in two broad bands, under and over four hours, so it could not corroborate the 20- to 30-minute attack length obtained at interview. It therefore functions as a measure of overall headache burden and treatment response rather than as a source of phenotypic data, and in a suspected TAC, an attack count would have been more informative than a day count. He still gets occasional mild headaches after poor sleep, which he regards as different from his old attacks; more than one headache disorder may have been present, and the diary may have counted both. Verapamil was never stopped, so there is no dechallenge and spontaneous remission cannot be excluded. Outcome was assessed by patient report over the telephone. No validated headache disability instrument, standardized pain score, prospective attack count, or objective measure of functional improvement was used at any point, and no electrocardiogram or follow-up cardiovascular observations were recorded before or during treatment. The neuroimaging was not available to us for independent review, so the vascular findings rest on the referring radiology report. Finally, we never saw the reasoning behind the original diagnosis of trigeminal neuralgia, so our account of diagnostic bias is a reconstruction rather than a documented sequence.

Patient perspective

The patient described the change in plain terms when we telephoned him. He said the attacks no longer occur and that the occasional mild headaches he still gets after poor sleep do not trouble him. He thanked us for the care he received and said that, as far as he is concerned, nothing could be better.

## Conclusions

This case illustrates a headache phenotype suggestive of paroxysmal hemicrania, comprising short-lasting, high-frequency, unilateral orbital and maxillary attacks accompanied by restlessness rather than cranial autonomic features, with cluster headache remaining a plausible alternative. It failed to respond to an adequate escalating indomethacin trial and therefore could not be classified as definite paroxysmal hemicrania. After verapamil was substituted, headache burden fell progressively over two months, ending in 18 consecutive headache-free days and sustained remission at follow-up. Whether verapamil produced that change cannot be established from a single uncontrolled case, as the drug was never withdrawn, and spontaneous remission remains possible. Two lessons follow. Failure to document an absolute indomethacin response should prompt reassessment of treatment adequacy, adherence, and the full differential diagnosis, rather than automatic confirmation or dismissal of a paroxysmal hemicrania-spectrum diagnosis. A diagnosis that rests on treatment response deserves the same scrutiny as one that rests on symptoms. Further case series are needed to clarify the place of verapamil in indomethacin-non-responsive PH phenotypes and to refine the diagnostic boundaries within the TACs.
